# Confronting needlestick and sharp injuries in healthcare: a decade of struggle and progress in a university teaching hospital

**DOI:** 10.1186/s12913-025-12345-0

**Published:** 2025-02-01

**Authors:** Shuk-Ching Wong, Lithia Lai-Ha Yuen, Germaine Kit-Ming Lam, Monica Oi-Tung Kwok, Crystal Yuen-Ki Siu, Bella Hong-Yan Li, Jonathan Hon-Kwan Chen, Simon Yung-Chun So, Kelvin Hei-Yeung Chiu, Vincent Chi-Chung Cheng

**Affiliations:** 1 Infection Control Team, Queen Mary Hospital, Hong Kong West Cluster, Pokfulam, Hong Kong Special Administrative Region China; 2 Department of Microbiology, School of Clinical Medicine, Li Ka Shing Faculty of Medicine, The University of Hong Kong, Pokfulam, Hong Kong Special Administrative Region China; 3 School of Nursing, Li Ka Shing Faculty of Medicine, The University of Hong Kong, Pokfulam, Hong Kong Special Administrative Region China; 4 Department of Microbiology, Queen Mary Hospital, Pokfulam, Hong Kong Special Administrative Region China

**Keywords:** Needlestick, Sharp, Injuries

## Abstract

**Background:**

Needlestick and sharp injuries (NSIs) among healthcare workers (HCWs) are a significant concern in infection prevention. This study analyzes the incidence and characteristics of NSIs at Queen Mary Hospital and assesses the effectiveness of targeted training programs for interns.

**Methods:**

A retrospective analysis was conducted on NSI reports from 2014 to 2023, focusing on episodes involving medical, nursing, allied health, and other staff. Personal coaching training programs for newly recruited interns were implemented in 2019 to enhance safety practices. NSI episodes were analyzed in relation to patient days and full-time equivalents (FTE).

**Results:**

During the study period, there was a mean of 464,118 patient days per year, with an average of 5,928 HCWs per year. A total of 1,076 NSI episodes were reported, resulting in a mean of 2.31 episodes per 10,000 patient days and 1.82 episodes per 100 FTE. Medical staff accounted for 502 episodes (47%), while nursing staff had 339 episodes (32%). The mean NSI episodes among medical staff were significantly higher than nursing staff per year, measured per 10,000 patient days (1.08 ± 0.21 vs. 0.73 ± 0.16, *p* = 0.001) and per 100 FTE (8.46 ± 1.95 vs. 1.62 ± 0.40, *p* < 0.001). Of the 1,076 episodes, 430 (40%) occurred in HCWs with less than 1 year of experience, with 278 (65%) attributed to interns, contributing to 26% of the overall NSI burden. Most incidents among interns occurred in wards (95%, 264/278), with 95% (252/264) involving hollow needles. Notably, 54% (149/278) involved safety-equipped devices, yet 94% (140/149) of these were not activated properly. Following targeted training, NSI rates among interns significantly decreased from 0.82 to 0.46 per 10,000 patient days (R^2^ = 0.977, *p* = 0.001) and from 6.84 to 3.40 per 100 FTE (R^2^ = 0.874, *p* = 0.020) from 2019 to 2023.

**Conclusions:**

The study highlights the ongoing issue of NSIs among HCWs, especially inexperienced interns. While training programs have shown promise in reducing NSI rates, the high incidence of safety devices not being effectively utilized underscores the need for continuous education and hands-on training to enhance safety practices and prevent NSIs in healthcare settings.

## Introduction

Needlestick and sharp injuries (NSIs) represent significant occupational hazards for healthcare workers (HCWs) and are a critical concern in healthcare settings worldwide. These injuries facilitate the transmission of bloodborne pathogens such as human immunodeficiency virus (HIV), hepatitis B virus (HBV), and hepatitis C virus (HCV), posing risks not only to HCWs but also to patients. Despite widespread awareness and the establishment of safety protocols, NSIs continue to be prevalent, highlighting a persistent gap between policy and practice. Recent systematic reviews and meta-analyses reveal that the overall incidence of NSIs is approximately 43%, derived from 113 studies involving over 520,000 HCWs from 2000 to 2020 [1]. This alarming statistic underscores the need for renewed efforts to mitigate these risks. One of the reasons for a high overall incidence of NSIs is due to improper clinical practice, including the frequent recapping of needles, inappropriate disposal practices, and a lack of access to safer needle devices [2]. Workplace culture and the lack of staff training and resource allocation play important roles in the high incidence of NSIs.

From an infection control perspective, the importance of addressing NSIs cannot be overstated. Effective prevention strategies are crucial not only for protecting HCWs but also for safeguarding patients from potential infections that can arise from these incidents. The implementation of comprehensive training programs and the promotion of a strong safety culture are essential steps toward reducing NSIs [3].

Despite the wealth of literature on NSIs, there remains a notable gap in studies focusing on HCWs in our specific geographical area over an extended period. This gap underscores the need for localized data to inform effective interventions tailored to the unique challenges faced by healthcare settings in our region. Additionally, the role of gender in NSI incidence is a significant factor that warrants exploration. Research has indicated that gender can influence the prevalence and nature of NSIs among HCWs [4, 5]. Therefore, our study not only documents trends, challenges, and successes associated with NSIs but also aims to assess the impact of gender on these injuries. By considering these dimensions, we hope to contribute to the existing literature on effective strategies for reducing NSIs and enhancing safety in healthcare environments.

## Material and methods

### Setting

This study was conducted at Queen Mary Hospital, a university-affiliated facility with a capacity of approximately 1,700 beds, located in the Hong Kong West Cluster and governed by the Hospital Authority. All HCWs, including medical staff, nursing staff, allied health professionals, and care-related support staff, are required to receive infection control training upon recruitment and undergo refresher training every 24 months, per hospital policy. The infection control training covers standard and transmission-based precautions and the safe handling of needles and sharps. In the event of NSIs involving HCWs, the wound should be washed thoroughly and immediately with soap and water, and they are required to attend the Accident and Emergency Department for further management. Furthermore, these incidents are reported to the infection control nurse (ICN) for counseling and performing root cause analysis for the NSIs.

### Report of healthcare worker with needlestick and sharp injuries

Upon receiving reports of NSIs among HCWs, the affected staff was counseled and interviewed by the ICN to collect information on the demographics of the affected staff, including personal identifiers, professional categories, ranks, and years of experience in this hospital. Additionally, the details of the NSI were obtained from the affected staff, such as the working environment, the nature of the needle or sharp involved (e.g., hollow or non-hollow needle, a surgical instrument, or glass), and the mode of injury. Information on the mode of injury includes whether the NSI occurred when the HCW was handling the needle or sharp (with or without the influence of others), when the needle or sharp was not in use, or when the HCW was passively injured by another person handling the needle or sharp. Further information on whether safety devices were available and properly activated at the time of NSI was also obtained during the interview.

The information was recorded on a standard form to facilitate follow-up measures by the ICN based on the risk assessment of individual events of NSIs. These measures include retrieving the immunization records of HCWs and, if warranted, coordinating serological testing for HIV, HBV, and HCV of the source patient (with consent). Depending on individual incidents, the ICN may follow up with HCWs regarding interval blood testing and monitor the outcomes of post-exposure prophylaxis.

### Analysis of healthcare worker with needlestick and sharp injuries

For the purpose of epidemiological analysis, each NSI episode reported from 2014 to 2023 was independently reviewed by two ICNs at the rank of registered nurse using a standardized electronic database that included the aforementioned parameters. If any discrepancies in data entry were identified, the NSI episode was further reviewed by two additional ICNs at the rank of advanced practice nurse to reach a consensus. For unresolved incidents, the co-leaders of the infection control team, comprising a senior nursing officer and an infection control officer, made the final decision. To protect staff confidentiality, personal identifiers of the HCWs were removed from the data entry.

The incidence of NSIs was presented as the number of episodes per 10,000 patient days per year, categorized by professional rank, as well as the number of episodes per 100 full-time equivalents (FTE) for each respective professional rank. The FTE of HCWs across different professional ranks was recorded as of March 31 of each year, according to the annual report of the Hospital Authority [6]. Data on patient days at Queen Mary Hospital was retrieved from the records office.

### Campaign to promote safe handling of needles and sharps

Campaign to promote safe handling of needles and sharps by infection control team was described and correlated with the trend of NSI episode at Queen Mary Hospital during the study period.

### Outcome of needlestick and sharp injuries

The source patients were traced and tested for serological markers of HBV, HCV, and HIV with their consent. HCWs with episodes of NSIs were monitored for the use of antiviral therapy as post-exposure prophylaxis for HIV. In addition, the outcomes of HBV, HCV, and HIV seroconversion were observed.

### Statistical analysis

One-way ANOVA test and Student’s *t-*test were used as appropriate. Trends of NSIs were analyzed using linear regression. A *p*-value of < 0.05 was considered statistically significant.

## Results

### Setting

At Queen Mary Hospital, the mean ± standard deviation (SD) of patient days per year from 2014 to 2023 was 464,118 ± 29,284. The mean ± SD number of HCWs during the study period was 5,928 ± 242. There were significant differences in the mean ± SD number of medical staff (595 ± 19), nursing staff (2,088 ± 117), allied health staff (697 ± 38), and other staff (2,548 ± 77) per year (*p* < 0.001, One-way ANOVA). When considering medical, nursing, and allied health staff as professional staff, the mean ± SD number of professional staff was significantly higher than that of other staff per year during the study period (3,380 ± 170 vs. 2,548 ± 77, *p* < 0.001).

### Analysis of healthcare worker with needlestick and sharp injuries

Between 2014 and 2023, a total of 1,076 episodes of NSIs were reported to the infection control team, with a mean ± SD of 108 ± 20 episodes per year. This is equivalent to an overall mean ± SD of 2.31 ± 0.35 episodes per 10,000 patient days and 1.82 ± 0.36 episodes per 100 FTE. Of the 1,076 episodes, there were 502 (47%) among medical staff, 339 (32%) among nursing staff, 22 (2%) among allied health staff, and 213 (20%) among other staff. The prevalence of NSIs was higher in women compared to men, with a female-to-male ratio of 1.9 (701 vs. 375). However, among allied health and medical staff, the female-to-male ratios were 0.7 (9 vs. 13) and 0.9 (232 vs. 270), respectively. In contrast, the female-to-male ratios of NSIs among nursing staff and other staff were found to be higher, at 6.1 (291 vs. 48) and 3.8 (169 vs. 44), respectively.

**Fig. 1:**
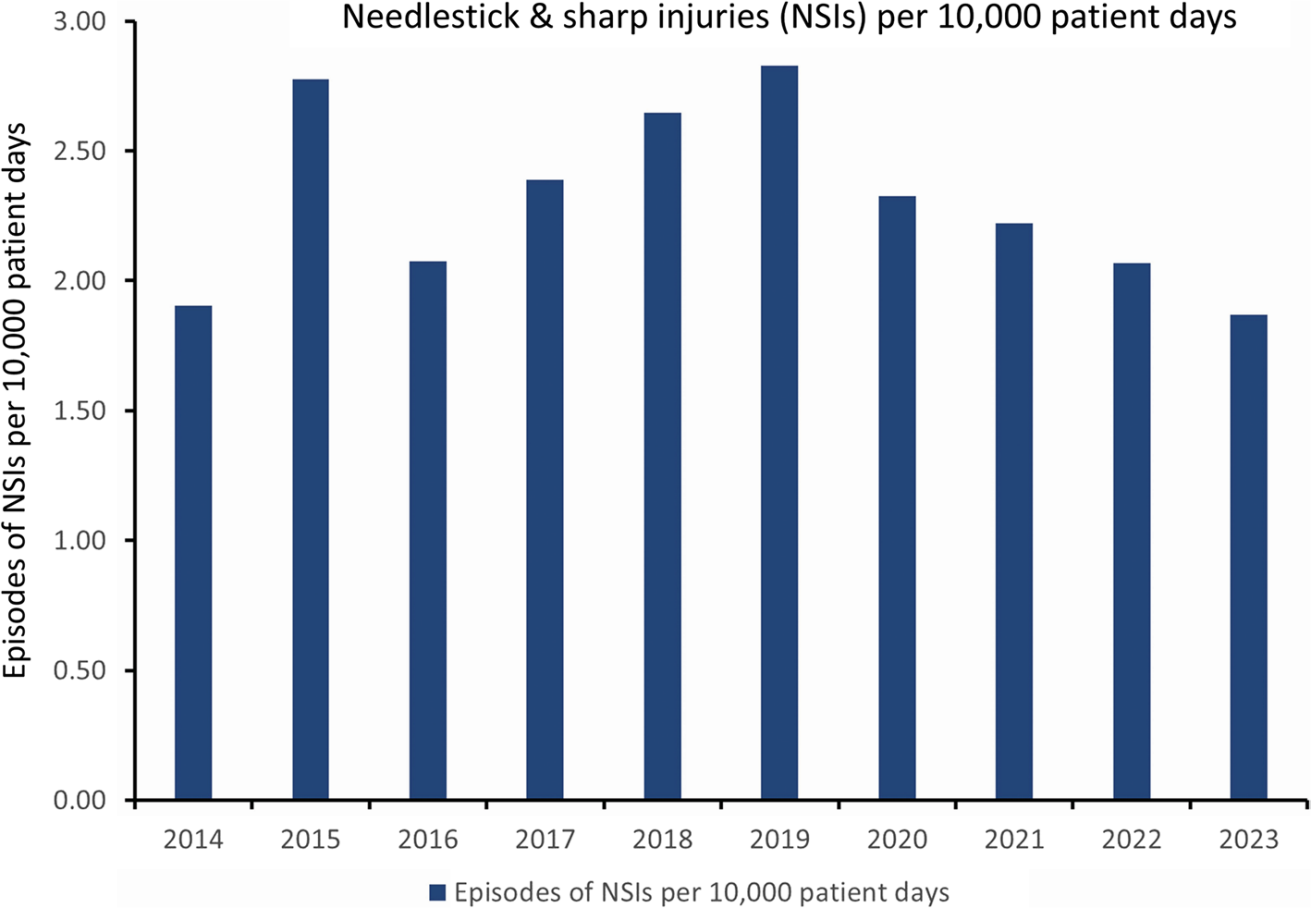
Trend of needlestick and sharp injuries per 10,000 patient days at Queen Mary Hospital. Note. NSIs, needlestick and sharp injuries

**Fig. 2:**
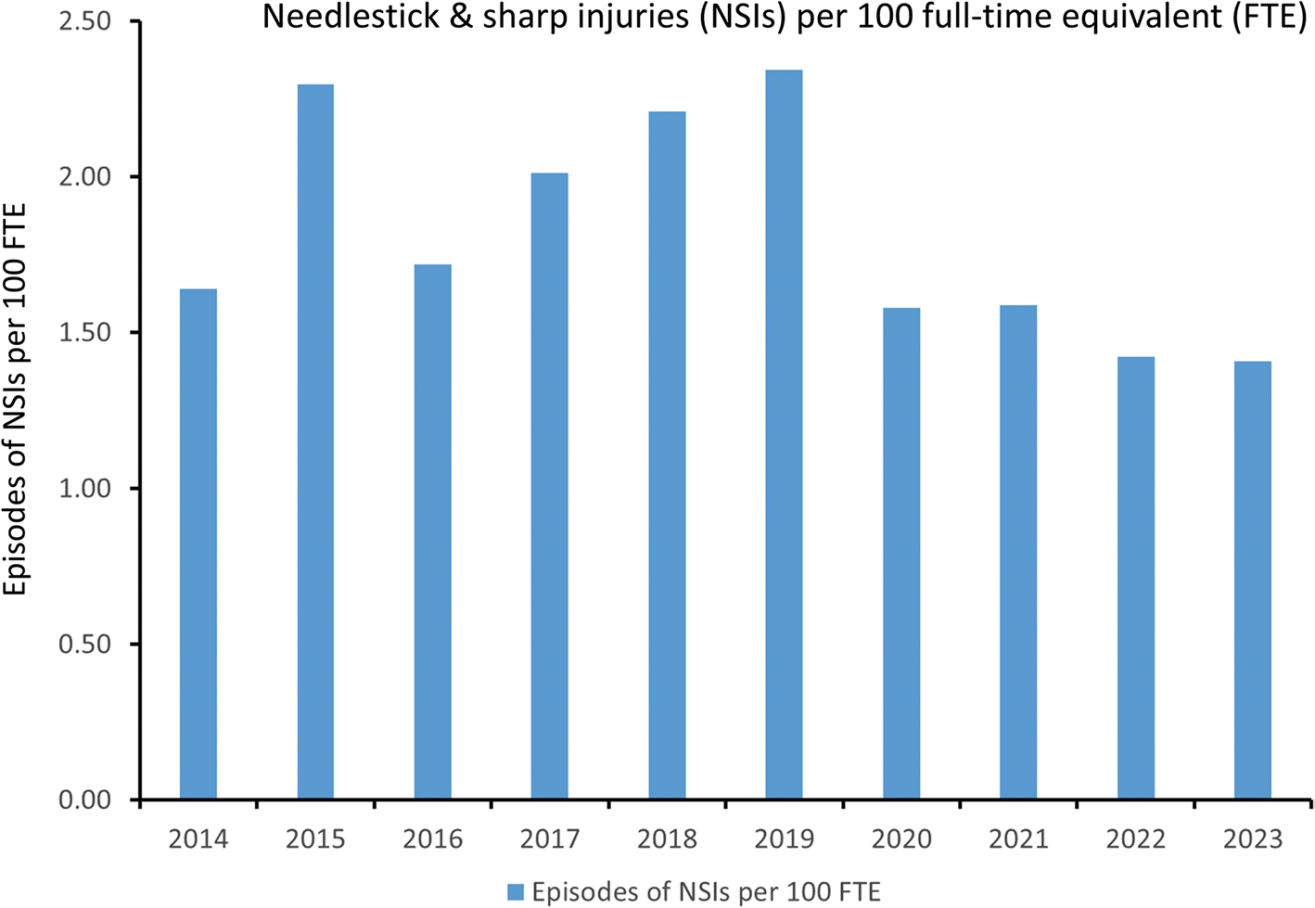
Trend of needlestick and sharp injuries per 100 full-time equivalents at Queen Mary Hospital. Note. NSIs, needlestick and sharp injuries; FTE, full-time equivalents

The ratio of NSI burden among medical staff to nursing staff was 1.5 (502/339). The mean ± SD of NSI episodes per 10,000 patient days across different staff categories was significantly different: medical staff (1.08 ± 0.21), nursing staff (0.73 ± 0.16), allied health staff (0.05 ± 0.04), and other staff (0.46 ± 0.17) (*p* < 0.001, One-way ANOVA). Similarly, the mean ± SD of NSI episodes per 100 FTE across different staff categories was also significantly different: medical staff (8.46 ± 1.95), nursing staff (1.63 ± 0.40), allied health staff (0.31 ± 0.24), and other staff (0.84 ± 0.36) (*p* < 0.001, One-way ANOVA). Specifically, the mean ± SD of NSI episodes among medical staff was significantly higher than that of nursing staff per year, measured per 10,000 patient days (1.08 ± 0.21 vs. 0.73 ± 0.16, *p* = 0.001), and per 100 FTE (8.46 ± 1.95 vs. 1.62 ± 0.40, *p* < 0.001). The trends of NSI episodes per 10,000 patient days and per 100 FTE are shown in Figs. 1 and 2, respectively.

**Fig. 3:**
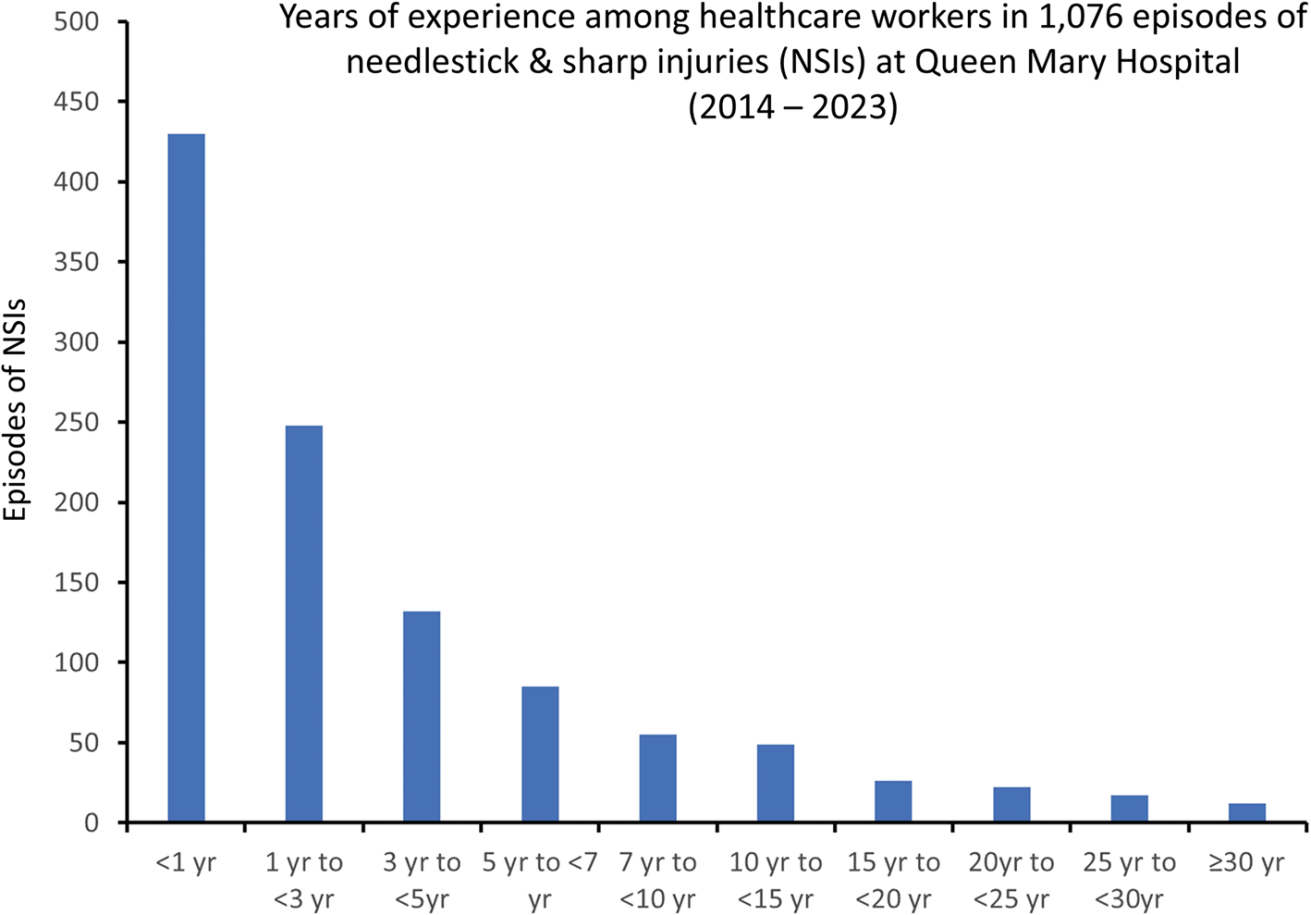
Years of experience among healthcare workers in 1076 episodes of needlestick & sharp injuries at Queen Mary Hospital (2014 – 2023). Note. NSIs, needlestick and sharp injuries

Of the 1,076 episodes of NSIs, 430 (40%) occurred in HCWs with less than 1 year of experience, while 248 (23%) occurred in HCWs with 1 to less than 3 years of experience, and 132 (12%) occurred in those with 3 to less than 5 years of experience (Fig. 3). The mean ± SD of NSI episodes per year across different experience groups was as follows: less than 1 year (43 ± 12), 1 to less than 3 years (25 ± 8), 3 to less than 5 years (13 ± 4), 5 to less than 7 years (9 ± 3), and 7 to less than 10 years (6 ± 3). These differences were statistically significant (*p* < 0.001, One-way ANOVA).

The majority of NSIs occurred in wards (59%, 630/1,076) and in the operating theatre (26%, 281/1,076). The mean ± SD of NSI episodes per year in wards (63 ± 12), operating theatre (28 ± 9), and other areas (17 ± 3) was significantly different (*p* < 0.001, One-way ANOVA). Hollow needles constituted the most common source of NSI (64%, 694/1,076), followed by surgical instrument (32%, 346/1,076). Non-hollow needles were associated with only 2% (18/1,076) of NSIs during the study period. The mean ± SD of NSI episodes was significantly higher for hollow needles compared to surgical instruments per year (69 ± 13 vs. 35 ± 10, *p* < 0.001).

Regarding the mode of injury, two ICNs reached a consensus assessment in 98% (1,057/1,076) of the episodes. The remaining cases were resolved by two advanced practice nurses in 17 out of 19 cases and by two co-leaders of the infection control team in 2 out of 19 cases, respectively. The NSIs occurred while HCWs were handling the needle or sharp in the following ways: with the influence of others (10%, 107/1,076), without the influence of others (61%, 652/1,076), when the needle or sharp was not in use (16%, 177/1,076), or when the HCW was passively injured by another person handling the needle or sharp (13%, 137/1,076). The mean ± SD of NSI episodes per year while HCWs were handling the needle or sharp, specifically, with the influence of others (11 ± 2), without the influence of others (65 ± 13), when the needle or sharp was not in use (18 ± 6), or when the HCW was passively injured by another person handling the needle or sharp (14 ± 5), showed significant differences (*p* < 0.001, One-way ANOVA).

**Fig. 4:**
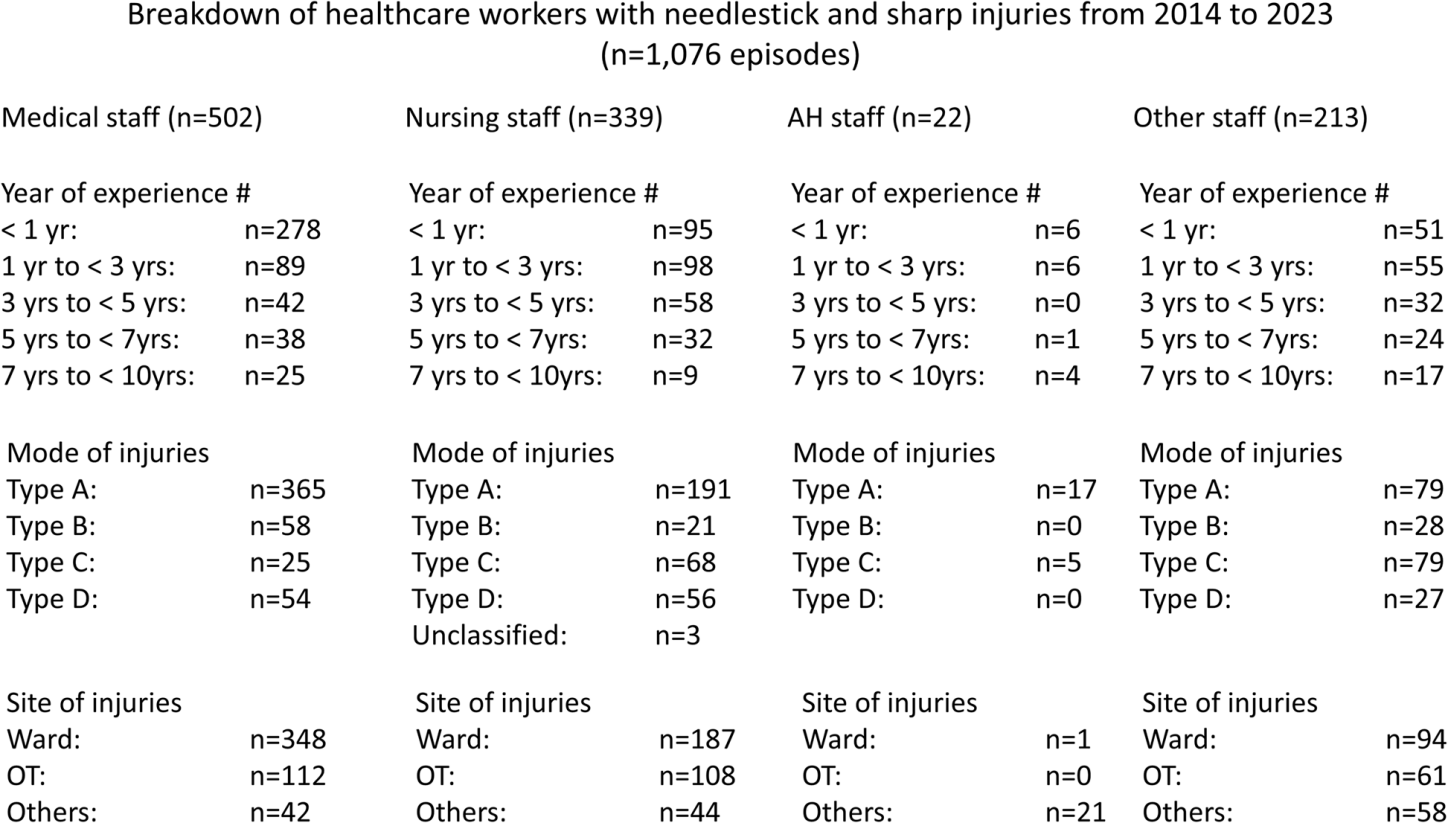
Breakdown of healthcare workers with needlestick and sharp injuries from 2014 to 2023. Note. # The data for healthcare workers with 10 or more years of experience is not shown. For the modes of injury, Type A represents needlestick and sharp injuries that occurred while healthcare workers (HCWs) were handling the needle or sharp without influence of others. Type B represents injuries that occurred while HCWs were handling the needle or sharp with the influence of others. Type C refers to needlestick and sharp injuries that occurred when the needle or sharp was not in use. Type D represents injuries where HCWs were passively injured by another person handling the needle or sharp

Of the 1,076 episodes of NSIs, 813 (76%) were associated with needles and sharps without safety features, while 263 (24%) were associated with needles and sharps with safety features. In the 263 NSIs associated with needles and sharps with safety features, 250 (95%) HCWs did not activate the safety mechanism during the handling of sharps. Breakdown of healthcare workers with needlestick and sharp injuries from 2014 to 2023 is shown in Fig. 4.

When comparing episodes of NSIs between HCWs with less than 1 year of experience and those with 1 year or more, a significantly higher proportion of medical staff experienced NSIs during their first year (65%, 278/430 vs. 35%, 224/646; *p* < 0.001). Moreover, HCWs with less experience sustained more NSIs while working in wards (81%, 348/430 vs. 44%, 282/646; *p* < 0.001) and while handling needles or sharps, both with the influence of others (14%, 61/430 vs. 7%, 46/646; *p* < 0.001) and without the influence of others (68%, 292/430 vs. 56%, 360/646; *p* < 0.001) (Table 1).

**Table 1:** Needlestick and sharp injuries among healthcare workers by years of experience

	Years of experience < 1 year (*n* = 430)	Years of experience ≥ 1 year (*n* = 646)	*P* value
HCWs: number (%) of episodes			
Medical staff	278 (64.7%)	224 (34.7%)	< 0.001
Nursing staff	95 (22.1%)	244 (37.8%)	< 0.001
Allied health staff	6 (1.4%)	16 (2.5%)	0.220
Other staff	51 (11.9%)	162 (25.1%)	< 0.001
Mode of injuries: number (%) of episodes			
Type A	292 (67.9%)	360 (55.7%)	< 0.001
Type B	61 (14.2%)	46 (7.1%)	< 0.001
Type C	45 (10.5%)	132 (20.4%)	< 0.001
Type D	32 (7.4%)	105 (16.3%)	< 0.001
Site of injuries: number (%) of episodes			
Ward	348 (80.9%)	282 (43.7%)	< 0.001
Operating theatre	55 (12.8%)	226 (35.0%)	< 0.001

For the modes of injury, Type A represents needlestick and sharp injuries that occurred while healthcare workers (HCWs) were handling the needle or sharp without the influence of others. Type B represents injuries that occurred while HCWs were handling the needle or sharp with the influence of others. Type C refers to needlestick and sharp injuries that occurred when the needle or sharp was not in use. Type D represents injuries where HCWs were passively injured by another person handling the needle or sharp

Of the 430 episodes of NSIs reported among HCWs with less than 1 year of experience, 278 (65%) were attributed to interns, contributing to 26% (278/1,076) of overall NSI burden during our study period. The majority of these incidents occurred in wards (95%, 264/278), with 95% (252/264) involving hollow needles. Among the 278 episodes, 54% (149/278) involved needles or sharps equipped with safety mechanisms, yet 94% (140/149) of these devices were not activated properly. In terms of the mode of injury, NSIs occurred while interns were handling needles or sharps with the influence of others (17%, 47/278) or without such influence (75%, 209/278). In addition, injuries occurred when the needles or sharps were not in use (4%, 10/278) or when interns were passively injured by another person handling the needles or sharps (4%, 12/278).

### Campaign to promote safe handling of needles and sharps

Since 2018, numerous measures have been implemented at Queen Mary Hospital for all HCWs, including staff education through the upload of educational materials to the infection control team website and the introduction of safer devices such as blood transfer devices, luer lock access devices, and heparinized syringes with safety features. Despite these efforts, the incidence of NSIs per 10,000 patient days and per 100 FTE increased from 2018 to 2019. A significant reduction in the overall incidence of NSIs was observed from 2.83 to 1.87 per 10,000 patient days (R^2^ = 0.915, *p* = 0.011) from 2019 to 2023, while the overall incidence per 100 FTE decreased from 2.34 to 1.41 (R^2^ = 0.688, *p* = 0.083), although this change was not statistically significant.

**Fig. 5:**
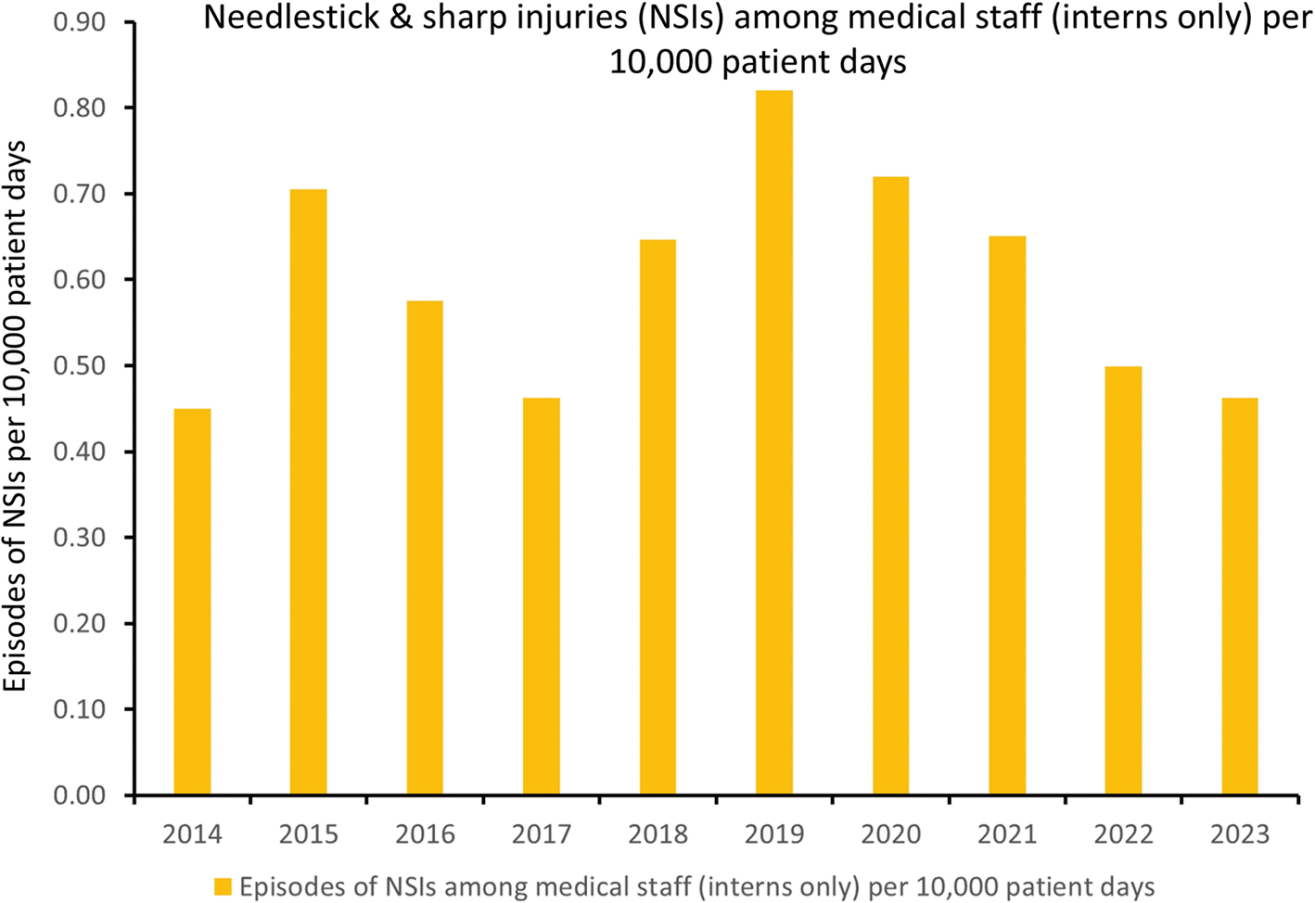
Needlestick and sharp injuries among medical staff (interns only) per 10,000 patient days at Queen Mary Hospital. Note. NSIs, needlestick and sharp injuries

Given that medical staff, particularly those with less than 1 year of experience, account for the majority of NSIs, special training sessions for all newly recruited interns in public hospitals have been conducted annually, centrally coordinated by the Hospital Authority. Additionally, since 2019, a designated training program with personal coaching has been implemented for interns at Queen Mary Hospital, led by the infection control team. This local program, coordinated by ICNs, includes both lectures and practical sessions. The ICNs emphasize to the newly recruited interns, supported by data, that medical interns are the most commonly affected group and contribute significantly to the hospital’s NSI burden. They provide individualized instruction on the proper use and disposal of various needles and sharps, demonstrating techniques one by one, along with the correct activation of safety devices where applicable. Each intern is required to perform return demonstrations to ensure they can manage needles and sharps safely. As a result, there has been a significant reduction in the incidence of NSIs among interns, decreasing from 0.82 to 0.46 per 10,000 patient days (R^2^ = 0.977, *p* = 0.001) (Fig. 5), and from 6.84 to 3.40 per 100 FTE (R^2^ = 0.874, *p* = 0.020) from 2019 to 2023.

### Outcome of needlestick and sharp injuries

Of the 1,076 episodes of NSIs, 1,001 (93%) were associated with an identifiable patient source, including 537 males. Among these, 11% (90/808) tested serologically positive for HBsAg, while 3% (24/804) and 0.4% (3/802) were positive for HCV Ab and HIV Ab, respectively. HCWs with these exposures were referred to designated clinics for follow-up. None of the HCWs reported seroconversion for HBV or HCV. For the 13 HCWs who received post-exposure prophylaxis against HIV, the clinical outcomes were not disclosed to the infection control team due to staff confidentiality protections.

## Discussion

Our study summarized the incidence and characteristics of NSIs among HCWs over 10 years, involving 1,076 reported episodes in a single-center experience at a tertiary care facility, with a mean of 464,118 patient days per year and an average of 5,928 HCWs during the study period. The absolute number of reported NSI episodes was notably higher than those found in previous single-center studies from the United States [7, 8], Germany [9], Israel [10], the Kingdom of Saudi Arabia [11], Iran [12], and Nepal [13], indicating that our data provides a substantial basis for the analysis. However, when analyzing the NSI episodes with respective to the number of patient days and manpower, our data showed a mean of 2.31 episodes per 10,000 patient days and 1.82 episodes per 100 FTE. These figures are lower than those reported in another 10-year study, which found a mean of 4.9 episodes per 10,000 patient days and 5.2 exposures per 100 FTE at a university teaching hospital in the United States [14]. In addition, our data are lower than those reported in a 2015 survey of trends in occupational practice involving 181 hospitals across 34 states, which indicated a rate of NSI of 2.1 episodes per 100 FTE [15].

The overall prevalence of NSIs was higher in women compared to men, which aligns with previous reports [1, 4, 5]. This gender difference may be attributed to the high female-to-male ratio of NSIs among nursing staff in our study. However, this disparity can also be explained by the overall gender ratio (males per 100 females), which was reported as 15.3 in the “2019 Health Manpower Survey on Registered Nurses” in Hong Kong [16]. For medical staff, the female-to-male ratio of NSIs was 0.9, slightly higher than the overall female-to-male workforce ratio of medical doctors, which was 0.5 at the end of 2016 [17]. The gender difference in NSIs deserves further investigation.

Significant differences in NSI incidence across staff categories suggest systemic issues within the healthcare environment. Our ratio of NSI burden among medical staff to nursing staff was 1.5, which is higher than the 1:1 ratio reported in 2023 in the EPINet Sharp Object Injury and Blood and Body Fluid Exposure Reports. Established in 1992 by the International Safety Center, this platform allows hospitals to voluntarily submit data on occupational exposures to blood and body fluids [18]. Our medical staff had five times more NSIs than that of nursing staff in terms of NSI episodes per 100 FTE. This finding aligns with previous reports indicating a higher incidence of NSIs among medical interns than nurses [19]. However, other studies frequently cited nursing staff as the most commonly affected group in various NSI studies [20–24]. This disparity may be due to the differences in responsibilities and clinical environments of medical and nursing staff in different hospitals. Medical staff frequently engage in invasive procedures that carry a higher risk of NSIs, while nursing staff have more opportunities to handle needles and sharps in various settings, such as administering medications and phlebotomy. The higher incidence among nursing staff may be attributed to their greater number of exposures to needles and the fast-paced nature of their work, which can lead to lapses in safety practices. In addition, the high patient-to-nurse ratio can increase workloads and distractions during usual procedures, further elevating the risk of NSIs.

The experience of HCWs remains an important determining factor for NSIs. Over 60% of NSI episodes occurred in HCWs with less than 3 years of experience. Notably, medical interns with less than one year of experience, accounted for 26% of the total NSI burden during our study period. Most incidents occurred in wards, primarily involving hollow needles. Alarmingly, while 54% of the episodes involved safety-equipped devices, 94% of these were not activated properly by the interns. NSIs predominantly occurred while handling needles or sharps, either with or without the influence of others. This aligns with previous studies indicating that inexperienced HCWs are at greater risk for NSIs due to a combination of lack of experience and adequate training in handling needles and sharps [19, 25]. These findings underscore the necessity for targeted educational interventions aimed at new staff, particularly interns, who are often less familiar with safety protocols. Therefore, we adopt a personal coaching approach similar to influenza vaccination promotion [26], by implementing targeted training programs. This approach has led to a significant reduction in NSI episodes in terms of 10,000 patient days as well as per 100 FTE from 2019 to 2023. This highlights the effectiveness of structured training sessions that provide detailed introductions to needles and sharps handling, as well as the proper activation of safety devices.

Interestingly, despite the implementation of safety devices and educational campaigns beginning in 2018, our data indicated an initial rise in NSIs. Typically, one would expect a decrease in NSIs following the introduction of needleless systems or devices with engineered sharps-injury protection [27–29]. This counterintuitive trend may be due to the need for time for the staff to learn how to use the safety devices properly. Alarmingly, 95% of NSIs involved safety devices among all HCWs that were not activated correctly, emphasizing the need for ongoing education and hands-on training to ensure proper utilization of safety features.

The majority of NSIs occurred in clinical wards (59%) and operating theatres (26%), consistent with findings from other studies that indicate these environments pose higher risks due to the nature of the tasks performed [30, 31]. Hollow needles were the most common source of NSIs (64%), followed by surgical instruments (32%). This underscores the importance of focusing safety training on the design and handling of hollow needles, as they represent a significant risk factor.

Promoting the safe use of needles and sharps presents several challenges, particularly due to the perception of minimal risk associated with NSIs. The literature indicates that the risk of HBV, HCV, and HIV seroconversion following an NSI is relatively low [32]. In our study, the seroprevalence of the source patients were 11% for HBV and 3% for HCV, which are consistent with previously published data in Hong Kong [33, 34]. We did not observe any HBV or HCV seroconversion among HCWs. However, the data on potential HIV seroconversion was unavailable for further analysis. In Hong Kong, if the source patient is found to be infected by HIV, the HCWs are referred to designated clinics instead of the infection control team of their own hospitals for further follow-up and management. The in-charge doctor at the designated clinic is required to make anonymous referrals to the Expert Panel on HIV Infection of Health Care Workers. This panel was formed in 1994 by the Department of Health in accordance with the recommendations of the Hong Kong Advisory Council on AIDS, primarily assesses and advises on the need for job modification of HIV-infected HCWs and conducts lookback investigations [35]. While maintaining confidentiality is essential, it limits the capacity to gather comprehensive data on the actual outcomes of NSIs and the effectiveness of implemented safety protocols in individual hospitals, hence it may hinder proactive measures to enhance safety training and compliance.

This study has several limitations that should be acknowledged. Firstly, it is a single-center study, which may limit the generalizability of the findings to other healthcare settings. The reliance on reported episodes of NSIs may also introduce reporting bias, as not all incidents may have been documented or reported to the infection control team. Additionally, while we gathered data on seropositivity among source patients, we were unable to track the long-term outcomes of HCWs referred for follow-up after exposure, particularly regarding HIV seroconversion, due to existing policies in Hong Kong. This lack of comprehensive follow-up data may hinder a full understanding of the implications of NSIs and the effectiveness of response measures. Lastly, the evolving nature of safety protocols and devices over the study period may impact the consistency of the data, underscoring the need for ongoing research to evaluate the effectiveness of interventions in various healthcare environments.

## Conclusions

The incidence of NSIs among HCWs remains a significant concern, particularly among those with less experience, such as interns. Ongoing educational initiatives and targeted training programs are essential for enhancing safety practices and the proper use of safety devices. The observed initial rise in NSIs following the implementation of safety measures underscores the necessity for continuous education and hands-on training to ensure effective utilization of these features. To foster a culture of safety, it is crucial to address the challenges posed by confidentiality regulations and the perceived minimal risk of NSIs. Continued monitoring and assessment of NSI trends will be vital for identifying areas needing improvement and ensuring a safer working environment for all healthcare professionals.

## Data Availability

The datasets generated for this study will be made available in anonymized form from the corresponding author upon reasonable request.
